# Prediction of TP53 mutations across female reproductive system pan-cancers using deep multimodal PET/CT radiogenomics

**DOI:** 10.3389/fmed.2025.1608652

**Published:** 2025-09-01

**Authors:** Tianming Du, Tao Jiang, Xuanyi Li, Md Mamunur Rahaman, Marcin Grzegorzek, Chen Li

**Affiliations:** ^1^College of Medicine and Biological Information Engineering, Northeastern University, Shenyang, China; ^2^College of Intelligent Medicine, Chengdu University of Traditional Chinese Medicine, Chengdu, China; ^3^Department of Radiology, Shengjing Hospital of China Medical University, Shenyang, China; ^4^The University of New South Wales, Sydney, NSW, Australia; ^5^Institute of Medical Informatics, University of Luebeck, Luebeck, Germany

**Keywords:** PET/CT, TP53, deep learning, endometrial cancer, ovarian cancer, cervical cancer

## Abstract

**Background:**

TP53 mutations play a critical role in the clinical management and prognostic evaluation of gynecologic malignancies such as cervical, endometrial, and ovarian cancers. With the advancement of radiomics and deep learning technologies, noninvasive AI models based on medical imaging have become important tools for assessing TP53 mutation status.

**Methods:**

This study retrospectively analyzed 259 patients with cervical, endometrial, or ovarian cancer who underwent PET/CT before treatment. Radiomics features from tumors and brown adipose tissue (BAT) were extracted, and a Transformer-based model was developed to predict TP53 mutation by integrating imaging and clinical data. The model was trained with five-fold cross-validation, and clustering analysis was performed on deep features to explore their correlation with TP53 status.

**Results:**

Radiomic features from tumor CT images, tumor PET images, brown adipose tissue CT images, and brown adipose tissue PET images were all found to be associated with TP53 mutation status in gynecological tumors. On the test set, the accuracy of the tumor CT radiomic model was 0.7931, the tumor PET radiomic model achieved an accuracy of 0.8276, the brown adipose tissue CT radiomic model had an accuracy of 0.7241, and the brown adipose tissue PET radiomic model reached an accuracy of 0.7931. The combined model achieved an accuracy of 0.8620 on the test set, and after automatic annotation using nn-UNet, the combined model’s accuracy was 0.8000. Unsupervised clustering of the deep features extracted by the combined model showed that the image clustering patterns were significantly correlated with TP53 mutation status (*p* = 0.001, *p* < 0.05), indicating that our model successfully captured TP53-related features that exist across different cancer types.

**Conclusion:**

This study demonstrates that radiomic features from tumor and brown adipose tissue CT and PET images are closely associated with TP53 mutation status in gynecological tumors. This study constructed a cross-cancer TP53 model. The combined model constructed based on multi-modal imaging effectively captures TP53-related imaging phenotypes across different cancer types, and these phenotypic patterns show a significant correlation with TP53 mutation status.

## 1 Introduction

Cervical, endometrial, and ovarian cancers are the three major malignancies of the female reproductive system, contributing to an estimated 5 million deaths globally each year (1). Cervical cancer accounts for approximately 3.1% of new cancer cases, while endometrial cancer constitutes 2.2%, and ovarian cancer makes up 1.6%. TP53, a tumor suppressor protein, acts as a key safeguard mechanism against cancer by inhibiting cell division and responding to various stresses. Therefore, TP53 tumor suppressor gene mutations frequently occur in human cancers (2). In cervical cancer, TP53 mutations can be attributed to HPV infection, rendering individuals susceptible to the disease (3). TP53 mutations in endometrial cancer aid in identifying specific, high-risk tumor genotypes/phenotypes. In endometrial cancer, the CNH subgroup includes all uterine serous carcinomas and approximately 25% of high-grade endometrial cancer, which exhibits TP53 pathogenic mutations. Besides its diagnostic role, TP53 serves as a predictive factor in chemotherapy (4). In high-grade ovarian cancer, TP53 mutations are widespread (5), and TP53 inactivation assessment can predict the intrinsic and acquired resistance to taxane-based drugs (6). Despite differences in the epidemiology, genetic risk, and tumor microenvironment of various cancers, TP53 mutations play essential roles in these three types of tumors.

TP53 mutation status in gynecologic cancers can be highly accurately detected using p53 immunohistochemistry (IHC) (7). However, as a highly specific diagnostic method, IHC requires additional procedures, increasing the workload for pathologists. Currently, 18F-FDG is the most commonly used radiotracer for PET/CT (Positron emission tomography/computed tomography) imaging of malignant tumors, with a primary focus on glucose metabolism within tissues. PET/CT offers advantages in initial staging and response assessment in cancer patients by combining functional and anatomical information (8). The diagnostic value of 18F-FDG PET/CT has been confirmed in various gynecologic tumors and has also been validated for assessing tumor responses to tumor markers and predicting patient responses to treatment (9–11). Researches suggests that there may be a highly similar growth pattern associated with TP53 mutations in different types of tumors in medical images from various modalities (12–14).

PET/CT not only provides imaging information of the tumor itself but also reflects relevant features of brown adipose tissue (15). Brown adipose tissue is a mitochondria-rich fat tissue whose main function is to regulate body temperature through non-shivering thermogenesis. It is abundant in newborns and, although present in smaller amounts in adults, is mainly distributed in areas such as the neck and supraclavicular region (16). As a metabolism-related biomarker, brown adipose tissue can predict weight loss and the risk of cancer cachexia in tumor patients (17). Moreover, the presence of brown adipose tissue and the browning of white adipose tissue exert certain anti-cancer effects by regulating tumor glycolytic metabolism (18). Studies have also shown that brown adipose tissue is closely associated with tumor mutation status and can serve as an independent risk factor to predict recurrence and mortality in tumor patients (19, 20). These findings suggest that brown adipose tissue holds broad application prospects in tumor-related research.

In the medical field, artificial intelligence methods have been extremely widely used (21). Medical images including pathology, pathological images, and radiology images are processed by artificial intelligence methods (22, 23). At present, multi-modal medical images based on gynecological tumors can predict the benign and malignant tumors, gene mutations, lymph node metastasis status, chemotherapy treatment response, patient prognosis and other types of medical information (24). Some of these techniques are already in practice. For example, deep learning-based classification of cells on cervical smears has been applied in medical practice to improve the efficiency of tumor screening (25–27).

This study extracted radiomic features from tumor regions and brown adipose tissue regions, analyzed their correlation with TP53 mutation status, and evaluated the distribution differences of these features among three major gynecological tumors—endometrial cancer, cervical cancer, and ovarian cancer. Based on this, we compared the radiomic features of tumor regions and brown adipose tissue regions under two modalities, PET and CT, and established four deep learning models to predict TP53 mutation status in these three tumors. Subsequently, a combined model was developed to predict TP53 mutation status. We further extracted the high-dimensional deep learning features from this model for clustering analysis.

## 2 Materials and methods

Our institutional review board has approved the current study (Shengjing Ethics Committee, 2023PS1013K). The written informed consent was waived for the retrospective cohorts.

### 2.1 Retrospective cohort

**Figure 1:**
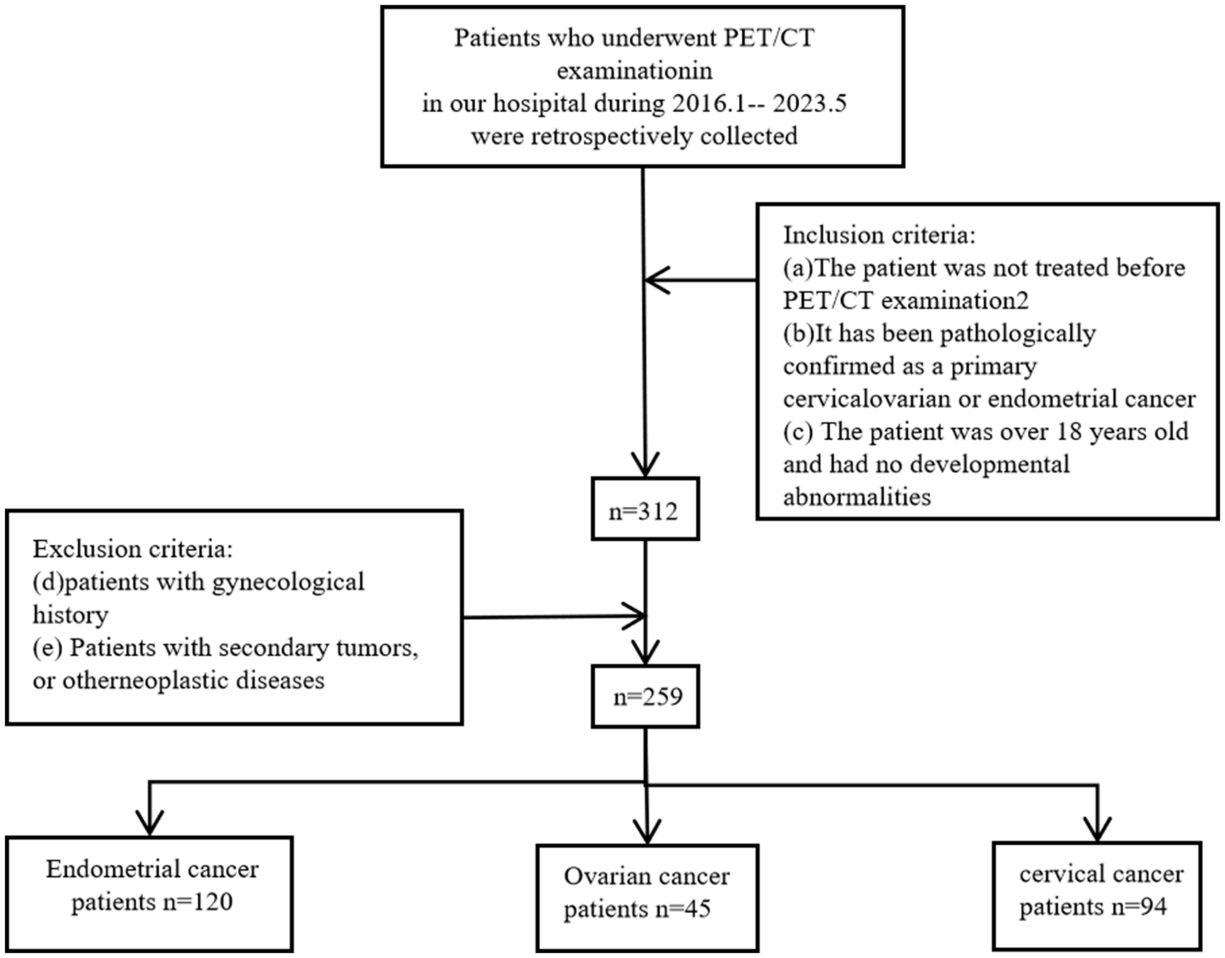
Inclusion and exclusion criteria workflow for patient data selection in cervical, endometrial, and ovarian cancers.

We conducted a retrieval of patients who visited Shengjing Hospital, China Medical University, from January 2016 to May 2023. As shown in Figure 1, we included patients according to the following criteria: (a) The patient underwent a PET/CT examination within 2 weeks of initial admission and received a biopsy or surgery to pathologically confirm any of primary cervical, endometrial, or ovarian cancer following the imaging examination; (b) Patients had not received any treatment before the PET/CT; and (c) Patients were over 18 years old and had no developmental abnormalities. Our study adhered to the following exclusion criteria; (d) Patients with a history of gynecological diseases; and (e) Patients with secondary tumors or a history of other tumors. As a result, this study included a total of 259 patients.

For privacy protection, we de-identified the data, retaining only images and relevant imaging information.

### 2.2 Pathology processing and TP53 assessment

Pathological specimens were obtained from the enrolled patients. Pathological specimens were prepared into H&E stained slides by standard procedure and photographed. All images were re-evaluated by pathologists to ensure that the central area of the images contained clearly identifiable typical tumor tissue. On this basis, the TP53 mutation status was determined using IHC. Paraffin-embedded sections were subjected to IHC staining, observed, and photographed under a microscope. The standard IHC SP method was used for susceptibility gene expression detection, and slides were examined by pathologists. Observations with staining greater than 5% were diagnosed as having a TP53 mutation. In addition, we also collected patients’ tumor staging, lymph node metastasis status, and pathological grading.

### 2.3 Radiomics and cross-modality tumor segmentation pipeline

Before 18F-fluorodeoxyglucose (18F-FDG) PET/CT scanning, all patients fasted for ≥6 h and had blood glucose levels of ≤7 mmol/L. They were injected with 3.70–5.55 MBq/kg 18F-FDG in the resting state. After 60 min, 18F-FDG PET/CT scanning was performed on a Discovery PET/CT 690 scanner (GE Healthcare, Waukesha, WI, USA) while the patients were lying on the patient bed. The scan ranged from the calvarium to the middle thigh (120 s/bed). The slice thickness, tube voltage, and tube current for CT scans were 3.75 mm, 120–140 kvp, and 80 mA, respectively.

**Figure 2:**
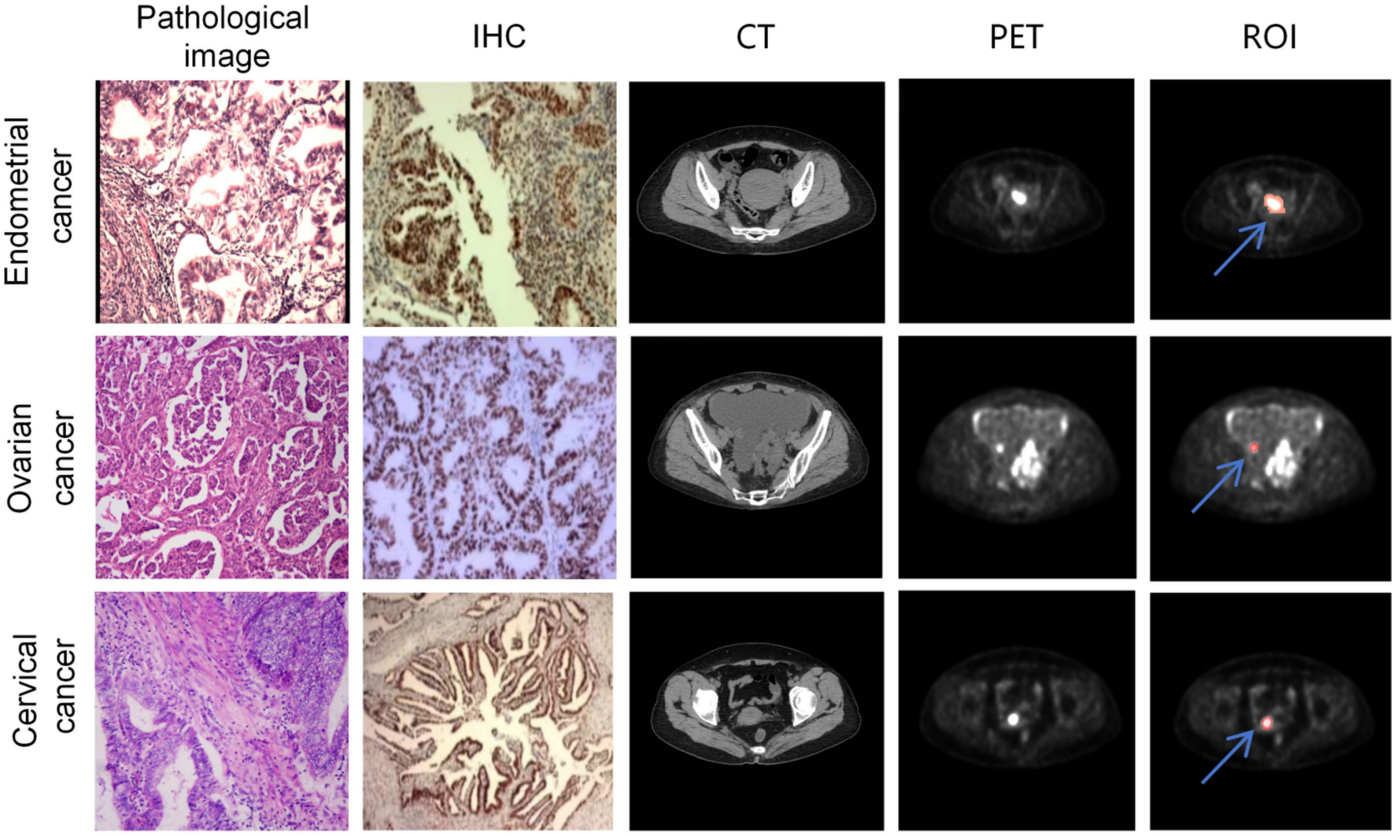
A sample consisted of the patient’s PET cross-sectional image containing the ROI. The first, second, and third rows represent samples from endometrial cancer, ovarian cancer, and cervical cancer, respectively. The first column shows the patient’s HE-stained pathological image, the second column displays the p53 immunohistochemistry stained image, the third column exhibits the cross-sectional CT image containing the tumor, the fourth column presents the cross-sectional PET image containing the tumor, and the fifth column indicates the ROIs annotated on the PET image (The red region marked by the blue arrow).

The image annotation was manually performed based on PET images using 3D Slicer by three radiologists with over 5 years of experience. The region of interest (ROI) was defined as the tumor area. In cases of disagreement among the radiologists, the final annotation was determined through consensus. Since PET and CT images were acquired on the same plane, the PET images were resampled to match the resolution of the CT images and then cropped to 512 × 512 pixels centered on the image. Both CT and PET images were normalized, as shown in Figure 2.

Radiomic features were separately extracted from tumor images of CT and PET, yielding a total of 1,781 features. All features were automatically extracted based on regions of interest (ROI) using the PyRadiomics tool. Feature extraction covered the original images as well as various filtered images, including square transformation (Square), square root transformation (SquareRoot), logarithmic transformation (Logarithm), exponential transformation (Exponential), Laplacian of Gaussian filtering (LoG), wavelet transform (Wavelet), 3D local binary pattern (LBP3D), and gradient images (Gradient). The final extracted features comprised 456 gray level co-occurrence matrix (GLCM) features, 342 first-order statistics features, 304 gray level run length matrix (GLRLM) features, 304 gray level size zone matrix (GLSZM) features, 266 gray level dependence matrix (GLDM) features, 95 neighboring gray tone difference matrix (NGTDM) features, and 14 shape-based features, which were used to quantitatively characterize the tumor imaging phenotypes from multiple dimensions.

To enhance the clinical usability of the diagnostic system, we developed a cross-modality gynecologic tumor segmentation model based on the annotated ROIs. We employed the nnU-Net framework for training (28). This framework is an adaptive benchmark segmentation method that automatically configures network architecture, preprocessing, training, and post-processing pipelines according to the characteristics of the dataset. We selected the 3D U-Net full resolution model structure and trained it for 100 epochs. During training, the model weights with the best validation Dice score were saved for each fold. At the testing stage, nnU-Net automatically enabled sliding window inference and incorporated test-time augmentation (TTA) to improve prediction stability. The final results were obtained by ensemble prediction using the five-fold models.

### 2.4 Identification and radiomics feature extraction of brown adipose tissue

We detected brown adipose tissue (BAT) activated under normal temperature conditions on PET/CT images. Regions with a standardized uptake value (SUV) greater than 1.5 were selected from the PET images, and combined with CT images, areas with CT values between −190 HU and −10 HU were identified as brown adipose tissue and annotated accordingly. Radiomics features were extracted separately from the BAT regions on both CT and PET images, yielding a total of 342 features. All features were automatically extracted based on regions of interest (ROIs) using the PyRadiomics tool. Feature extraction covered the original images and various filtered images, including square, square root, logarithm, exponential transforms, Laplacian of Gaussian (LoG), wavelet transform, 3D local binary pattern (LBP3D), and gradient images. For brown adipose tissue, only first-order statistical features were extracted to quantify the basic imaging properties of the fat regions through simple and intuitive pixel intensity distribution metrics, such as mean gray level, intensity range, and dispersion. This approach reflects the overall density and uniformity of the adipose tissue, facilitating the assessment of metabolic or structural differences, while avoiding the complexity and computational burden associated with higher-order radiomics features.

### 2.5 Deep learning-based TP53 classification model

**Figure 3:**
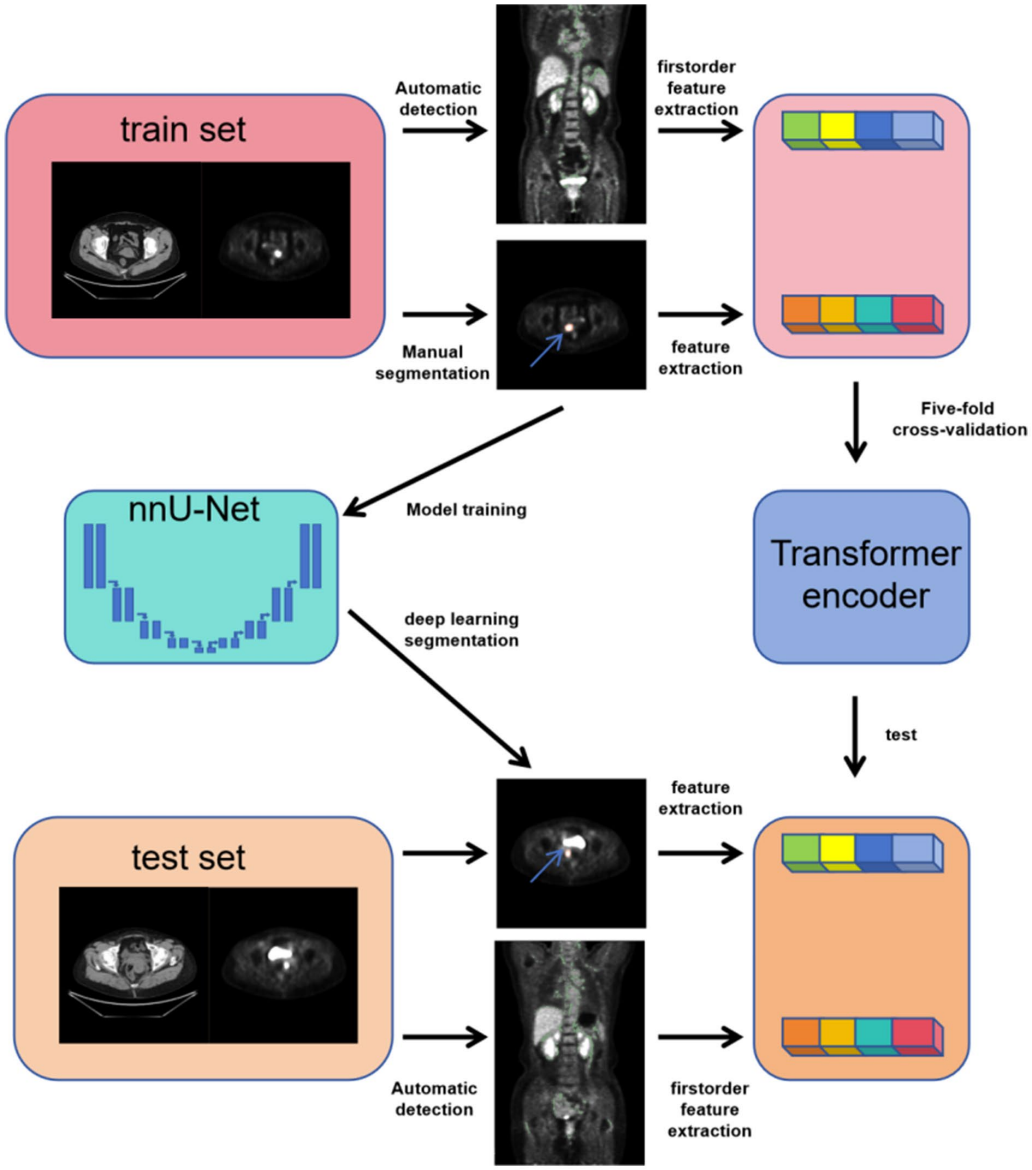
The flowchart illustrates the experimental design of this study.

As shown in Figure 3, in this study, we designed and implemented a multimodal feature modeling framework based on the Transformer architecture to predict the TP53 gene mutation status in three types of gynecologic tumors: cervical cancer, ovarian cancer, and endometrial cancer. The dataset was split into training and testing sets at a ratio of 9:1 to ensure the independence of model training and evaluation. During the training phase, five-fold cross-validation was further applied within the training set to optimize the model’s hyperparameters and assess its stability and generalization capability. In the cross-validation process, each fold’s model was trained on the remaining four folds and validated on the current fold, and based on this, the performance was compared on the test set. This approach fully leverages the limited sample size, improving the model’s adaptability and robustness in real-world applications. We modeled radiomics features from tumor CT images, tumor PET images, as well as CT and PET radiomics features of brown adipose tissue to evaluate their predictive power for TP53 mutation. Furthermore, for the combined model, we integrated pathological information reflecting patients’ biological characteristics, such as lymph node metastasis, tumor grading, and staging. By jointly inputting these heterogeneous multimodal data into the Transformer encoder, the model can thoroughly explore the latent relationships among different modalities, enhancing its ability to discriminate TP53 mutation status.

### 2.6 Feature clustering analysis

Based on the well-trained deep learning model, we extracted deep learning feature vectors from all samples. Hierarchical clustering was employed for clustering patient feature vectors (29). We used the agglomerative clustering method. We evaluated the clustering performance of the model using the Davies-Bouldin Index (DBI) to determine the optimal number of clusters and, consequently, the clustering pattern of image features (30). Given the characteristics of agglomerative clustering and for correlation analysis, we limited the number of clusters to a range of 2–9.

### 2.7 Statistical analysis

We evaluated the accuracy of the deep learning model for predicting TP53 mutation status. Additionally, we computed specific performance metrics, including Precision, Specificity, Sensitivity (Recall), and F1-score.

Statistical analysis was carried out using SPSS 26.0 software. Parametric data following a normal distribution were analyzed using t-tests or analysis of variance. Non-normally distributed data were tested using the Kruskal-Wallis H test. Group data were analyzed using the chi-square test. Pearson correlation analysis was used as well. Inter-group pairwise comparisons were performed using the Mann–Whitney U rank-sum test. A significance level of *p* < 0.05 was considered statistically significant. We separately assessed the correlations between TP53 mutation status and clustering, as well as cancer type and clustering.

## 3 Results

### 3.1 Baseline characteristics of patients

A total of 259 Endometrial Cancer, Ovarian Cancer and Cervical Cancer patients were included in this study, with their baseline characteristics, including age, type of cancer, TP53 mutations, BMI, Lymph node metastasis, tumor grade, as shown in Table 1.

**Table 1:** Clinical information of the samples.

		Endometrial cancer	Ovarian cancer	Cervical cancer
FIGO stage	I	83	2	57
	II	15	1	21
	III	11	29	14
	IV	4	12	0
	Indeterminate	7	1	2
Age		55 (50, 60.25)	55 (50, 62)	53.5 (46, 59)
BMI		24.80 (23.05, 27.55)	23.51 (21.23, 26.18)	22.66 (21.00, 24.79)
Lymph node metastasis	Positive	10	20	15
	Negative	110	25	79
Tumor grade	Well-differentiated	19	1	20
	Moderately differentiated	30	0	62
	Poorly differentiated	71	44	12

**Figure 4:**
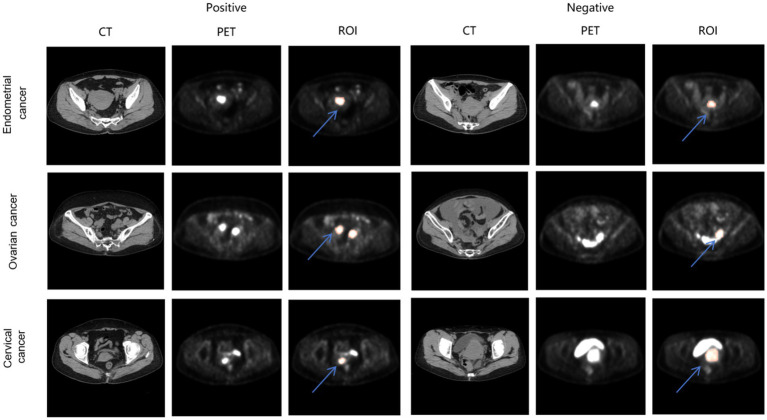
PET/CT images of endometrial cancer, cervical cancer, ovarian cancer in different TP53 states. The red region indicated by the blue arrow represents the annotated ROIs.

PET/CT images of different TP53 states in the three kind of tumors are shown in Figure 4.

### 3.2 Correlation analysis between radiomics features and TP53 status

**Figure 5:**
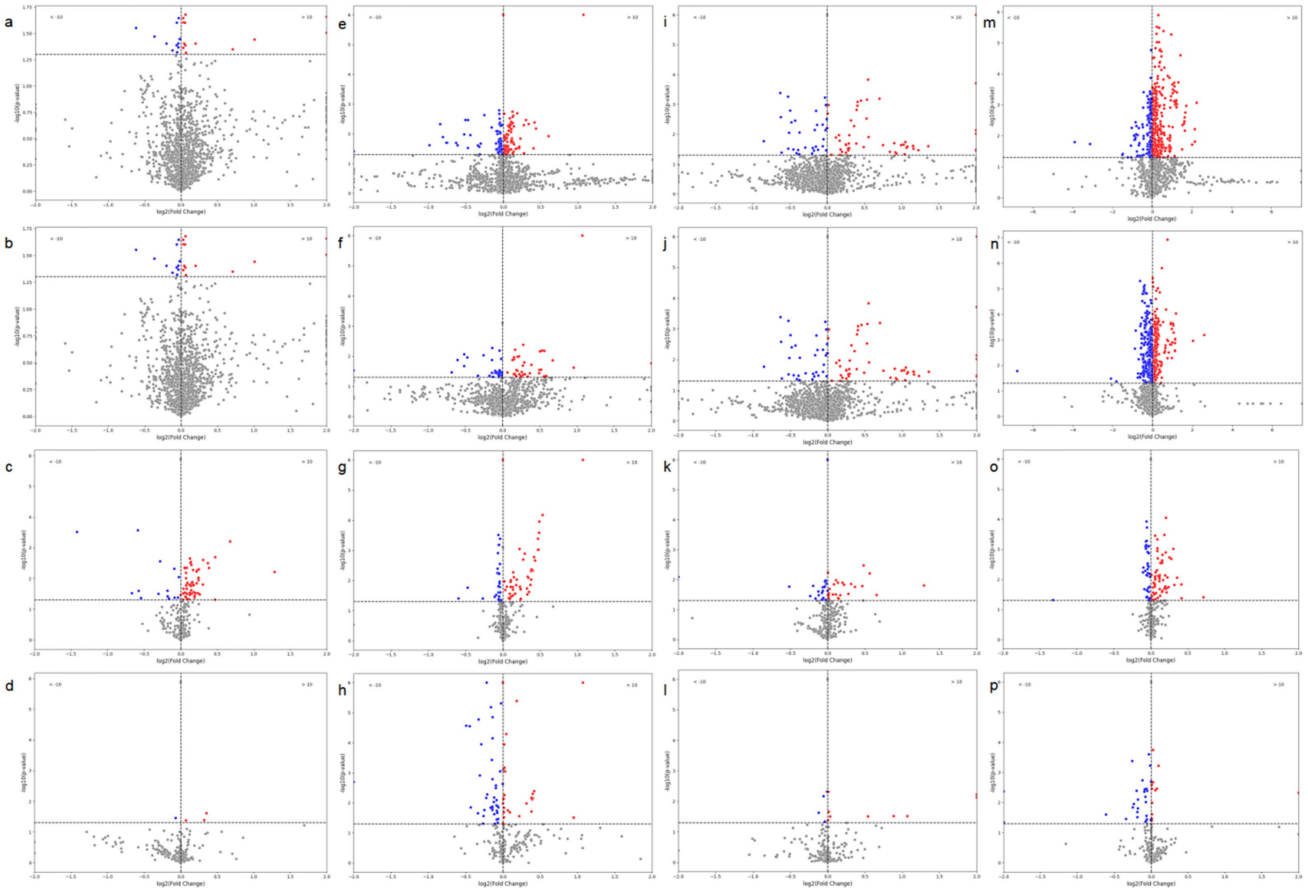
Volcano plots of radiomic features based on tumor images and brown adipose tissue (BAT) in relation to TP53. **(a)** CT-based radiomic features of tumors in ovarian cancer patients; **(b)** PET-based radiomic features of tumors in ovarian cancer patients; **(c)** CT-based radiomic features of BAT in ovarian cancer patients; **(d)** PET-based radiomic features of BAT in ovarian cancer patients; **(e)** CT-based radiomic features of tumors in endometrial cancer patients; **(f)** PET-based radiomic features of tumors in endometrial cancer patients; **(g)** CT-based radiomic features of BAT in endometrial cancer patients; **(h)** PET-based radiomic features of BAT in endometrial cancer patients; **(i)** CT-based radiomic features of tumors in cervical cancer patients; **(j)** PET-based radiomic features of tumors in cervical cancer patients; **(k)** CT-based radiomic features of BAT in cervical cancer patients; **(l)** PET-based radiomic features of BAT in cervical cancer patients; **(m)** CT-based radiomic features of tumors in all three cancer types; **(n)** PET-based radiomic features of tumors in all three cancer types; **(o)** CT-based radiomic features of BAT in all three cancer types; **(p)** PET-based radiomic features of BAT in all three cancer types.

We first performed differential analysis of radiomics features between TP53-negative and TP53-positive patients, calculating the statistical significance (*p*-values) and effect sizes for each feature. As shown in panels a–i of Figure 5, to explore the differences in the association between radiomics features and TP53 mutation status across different tumor types, we conducted feature differential analyses separately in patients with cervical cancer, ovarian cancer, and endometrial cancer. Volcano plots were used to visualize the statistical significance (*p*-values) and fold changes of each radiomic feature under different TP53 statuses for each tumor type, facilitating comparison of their performance differences across tumor types.

The results showed that although TP53-related radiomics features varied to some extent among the three tumor types, as shown in panels m–p of Figure 5, when cervical cancer, ovarian cancer, and endometrial cancer data were integrated for unified analysis, significant differences in tumor structural features (CT radiomics features) and metabolic features (PET radiomics features) were still observed between the TP53-negative and TP53-positive groups. Additionally, the structural features (CT features) and metabolic features (PET features) of patients’ brown adipose tissue were also closely associated with TP53 mutation status.

### 3.3 Model performance evaluation

For comparison, we constructed four models based on four different data sources: CT-based radiomic features of tumors, PET-based radiomic features of tumors, CT-based radiomic features of brown adipose tissue, and PET-based radiomic features of brown adipose tissue. Each model was evaluated using five-fold cross-validation. Subsequently, we incorporated all four types of features along with pathological features into a unified model. Additionally, we applied our custom tumor annotation using a segmentation model based on nn-UNet and conducted further testing on this basis.

#### 3.3.1 Model based on tumor radiomic features

**Figure 6:**
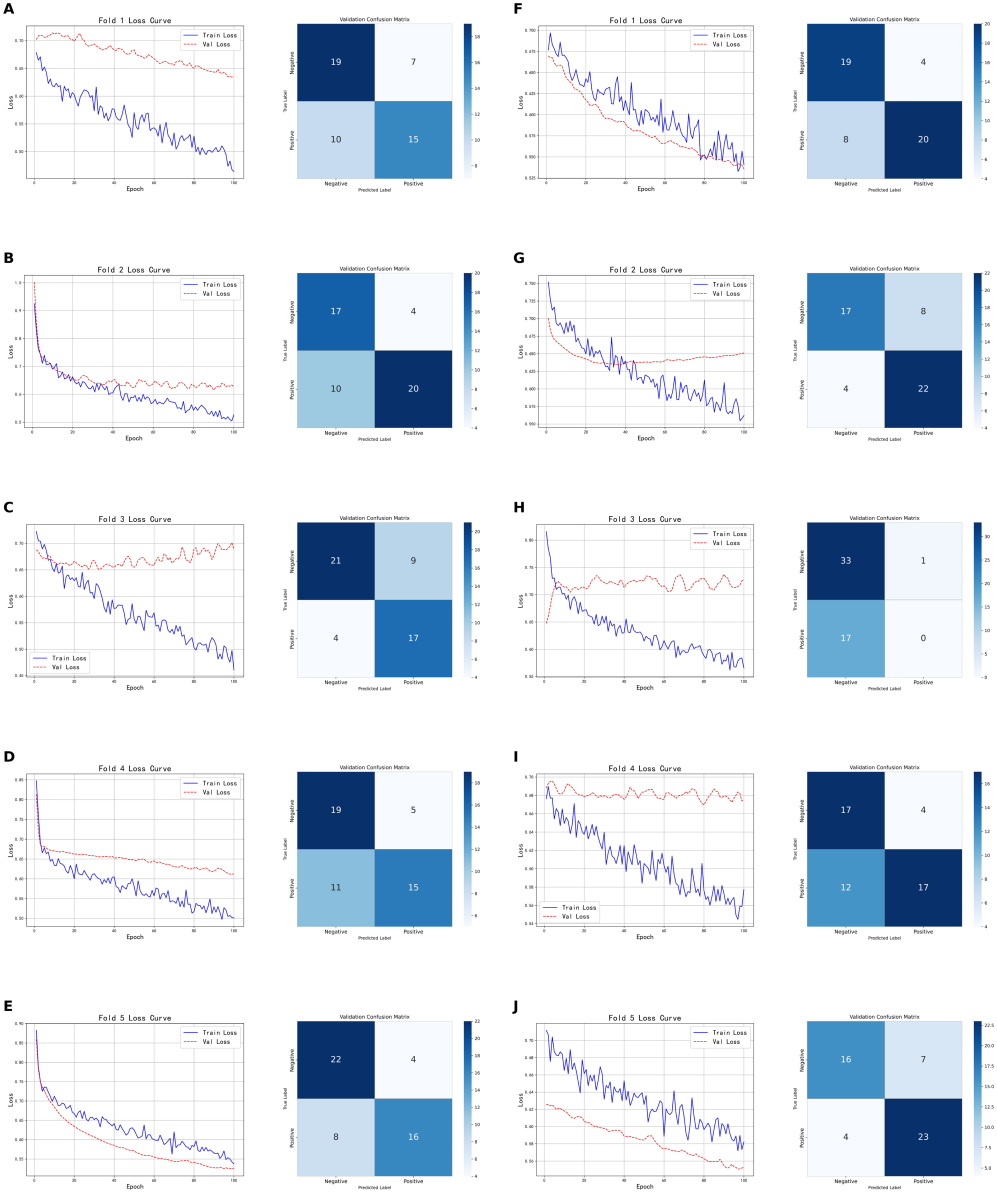
Five-fold cross-validation results of classification models based on tumor radiomic features. **(A–E)** Show the training and validation loss curves, as well as the confusion matrices on the validation sets, for each fold of the CT-based radiomic model. **(F–J)** Show the training and validation loss curves for each fold of the PET-based radiomic models, as well as the corresponding confusion matrices on the validation sets.

As shown in Figure 6, the models based on tumor data exhibited inconsistent performance across the five-fold cross-validation, both for CT-based and PET-based models. The model based on CT radiomic features achieved an accuracy of 0.7600 on the validation set, with a precision of 0.8000, recall of 0.6667, and F1-score of 0.7273. On the test set, it achieved an accuracy of 0.7931, precision of 0.8462, recall of 0.7333, and F1-score of 0.7857. In contrast, the model based on PET radiomic features performed better on the validation set, with an accuracy of 0.7800, precision of 0.7667, recall of 0.8519, and F1-score of 0.8070. It also demonstrated superior performance on the test set, with an accuracy of 0.8276, precision of 0.8462, recall of 0.7857, and F1-score of 0.8148. Overall, the model constructed using PET radiomic features outperformed the CT-based model in terms of classification performance, particularly in recall and F1-score, indicating a better capability in identifying positive samples.

#### 3.3.2 Models based on brown adipose tissue radiomic features

**Figure 7:**
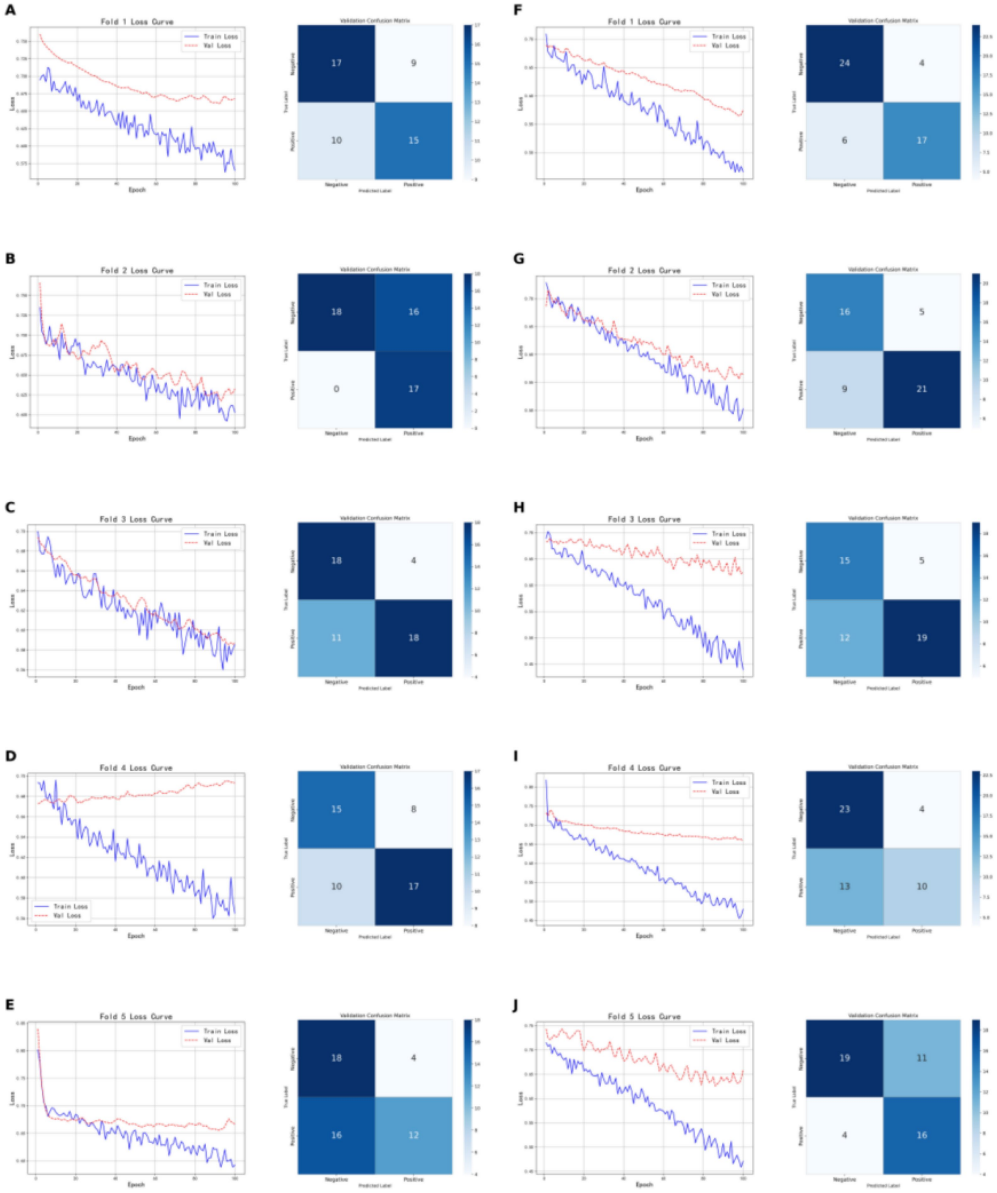
Shows the five-fold cross-validation results of classification models based on brown adipose tissue radiomic features. **(A–E)** Present the training and validation loss curves for each of the five folds of the CT-based radiomic models, along with the confusion matrices on the validation sets. **(F–G)** Show the training and validation loss curves for each fold of the PET-based radiomic models, as well as the corresponding confusion matrices on the validation sets.

As shown in Figure 7, the models based on brown adipose tissue data demonstrated greater stability across the five-fold cross-validation compared to models built on tumor features, for both CT-based and PET-based models. The CT radiomic feature model achieved an accuracy of 0.7059, precision of 0.8182, recall of 0.6207, and F1-score of 0.7059 on the validation set; on the test set, the accuracy was 0.7241, with both precision and recall at 0.7333, and an F1-score of 0.7333. In contrast, the PET radiomic feature model performed better on the validation set, with an accuracy of 0.8040, precision of 0.8095, recall of 0.7391, and F1-score of 0.7727; on the test set, it achieved an accuracy of 0.7931, precision of 0.8333, recall of 0.7143, and F1-score of 0.7692. Overall, the model constructed using PET radiomic features outperformed the CT model across all evaluation metrics, particularly showing clear advantages in accuracy and F1-score on the validation set, indicating better overall performance and stability in classification tasks based on brown adipose tissue features.

#### 3.3.3 Evaluation of the combined model

**Figure 8:**
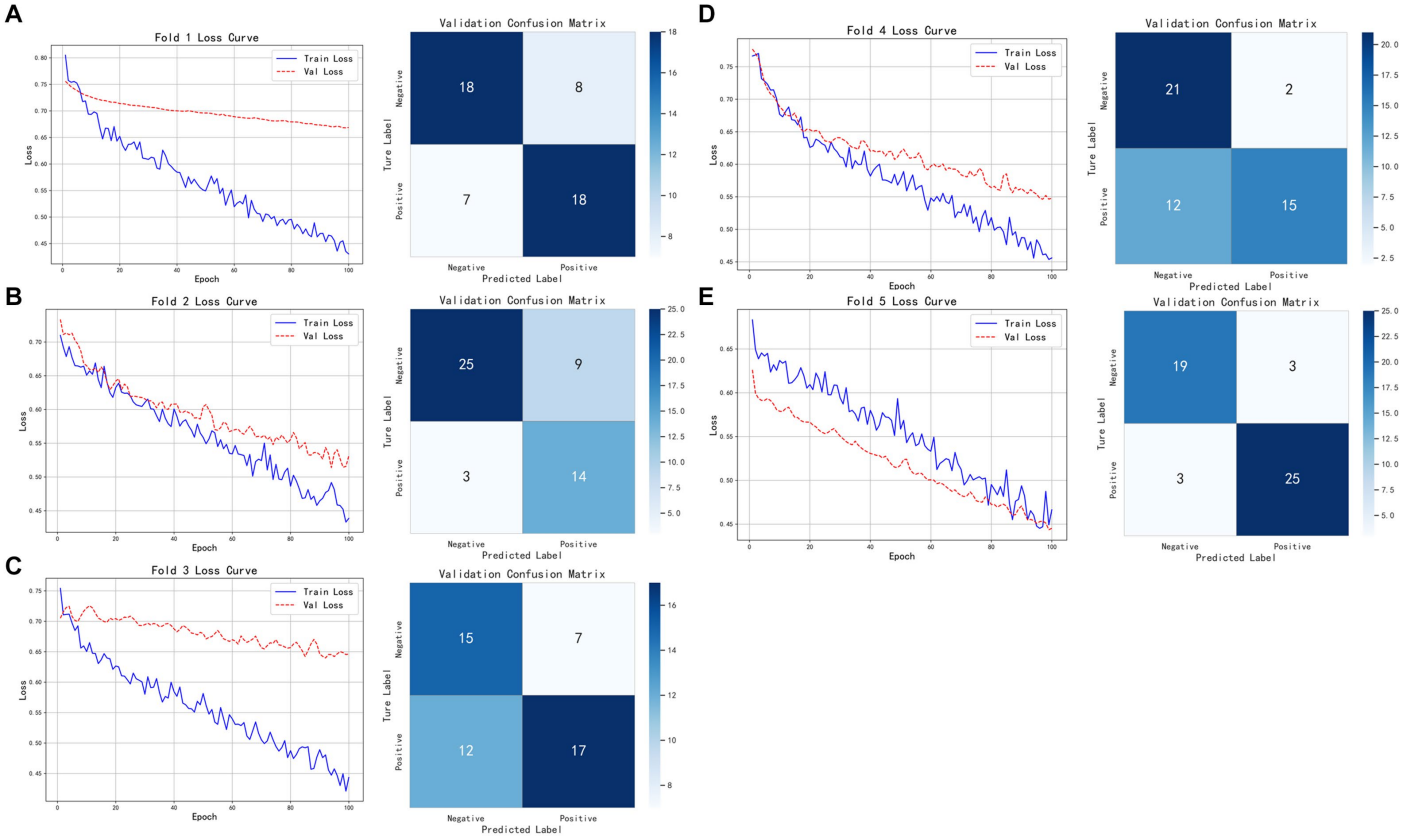
Five-fold cross-validation results of the classification model based on tumor imaging radiomic features, brown adipose tissue imaging radiomic features, and pathological features. **(A–E)** Show the training and validation loss curves for each of the five folds.

As shown in Figure 8, the model based on tumor data demonstrated relatively consistent performance across the five folds of cross-validation. The model’s training and validation losses closely tracked each other, indicating near-synchronous fitting. On the validation set, the model achieved an accuracy of 0.8800, with Precision, Recall, and F1-score all at 0.8929, demonstrating consistent and excellent classification performance. On the test set, the model attained an accuracy of 0.8620, Precision of 0.8235, Recall of 0.9333, and F1-score of 0.8750, indicating that while maintaining high accuracy, the model also has strong recognition ability, particularly excelling in recall and effectively identifying positive samples. Overall, the model shows good generalization ability and robust classification performance.

Building on this, we employed nn-UNet for automatic tumor annotation and then tested the classification model. The model achieved an accuracy of 0.8000, Precision of 0.7885, Recall of 0.8200, and F1-score of 0.8040 on the test set. The model’s performance under automatic annotation was similar to that with manual annotation. Although slightly lower in Recall and F1-score, it maintained a good balance between precision and recall. In summary, the model with nn-UNet automatic annotations still exhibits good classification capability and strong robustness while maintaining high accuracy.

### 3.4 Feature clustering and analysis

**Figure 9:**
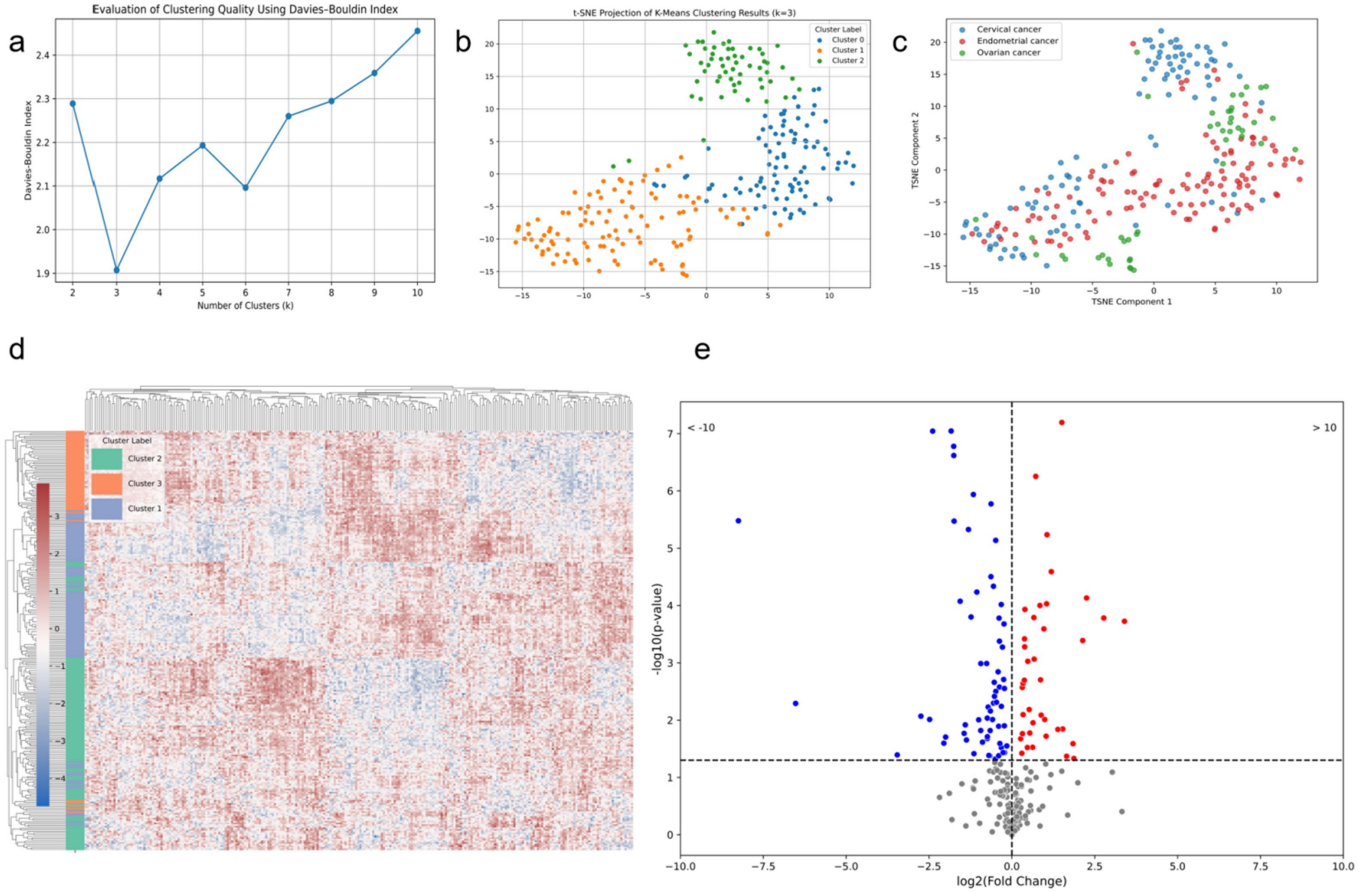
Modeling results based on deep features extracted from the combined model. **(a)** DBI corresponding to different numbers of clusters for the deep learning features; **(b)** t-SNE scatter plot with the number of clusters set to 3; **(c)** t-SNE scatter plot of different tumor types; **(d)** heatmap of deep features for the three clusters based on t-SNE; **(e)** volcano plot of deep features and TP53 mutation status.

After applying t-SNE dimensionality reduction to the features extracted by the deep learning classification model, we performed unsupervised clustering analysis, as shown in Figure 9a. When the number of clusters was set to three, the Davies-Bouldin Index (DBI) reached its minimum and was significantly lower than that for other cluster numbers, indicating the presence of three image patterns related to TP53. The t-SNE reduced clustering results of these patterns are shown in Figures 9b, c. Correlation analysis revealed a significant association between the image clustering patterns and TP53 mutation status (*p* = 0.001, *p* < 0.05), demonstrating that our model effectively captured TP53-related features across cancer types. As shown in Figure 9d, the clustering results after t-SNE reduction were nearly identical to those before reduction, further indicating that the deep features extracted by the model already had strong discriminative ability in the original feature space. Figure 9e shows that a considerable number of these deep features are directly related to TP53 mutation status.

## 4 Discussion

TP53 mutations are prevalent in gynecological tumors and have a significant impact on patient prognosis (31). Studies have shown that in gynecological tumors, TP53 mutation status can be predicted using data from various modalities (32, 33). In this study, we demonstrated the correlation between TP53 mutation status and tumor-related CT and PET features, as well as brown adipose tissue-related CT and PET features in patients with gynecological tumors. Based on this, we predicted TP53 status separately using tumor CT features, tumor PET features, brown adipose tissue CT features, and brown adipose tissue PET features, finding that models based on PET features performed better. Subsequently, we developed a combined prediction model and proposed a fast delineation method based on nnU-Net, both achieving good results on the test set. Finally, we analyzed the deep features extracted by the combined model, further confirming that our model successfully captured cross-modal TP53 imaging features.

Our study demonstrates a significant association between TP53 mutation status and various radiomic features, which is evident across different tumor types. Although there is some heterogeneity in how radiomic features respond to TP53 mutations in cervical, ovarian, and endometrial cancers, integrative analysis revealed a shared imaging feature pattern across these cancer types. This unified pattern likely reflects TP53’s role as a key tumor suppressor gene regulating common biological processes—such as cell cycle control, DNA repair, and metabolic reprogramming—across different gynecological tumors, thereby manifesting as recognizable imaging features in structural and metabolic scans (34). Brown adipose tissue (BAT) is recognized for its critical role in metabolism and has shown potential when combined with deep learning approaches (35, 36). BAT has been linked to tumor mutation characteristics and is considered directly related to tumor activity (37–39). In our study, we found that CT and PET features of brown adipose tissue in patients are also significantly associated with TP53 mutation status. Previous studies have shown that browning of subcutaneous adipose tissue can contribute to weight loss and cancer-associated cachexia. Additionally, thermogenic adipocytes locally activated within the tumor microenvironment may accelerate cancer progression by supplying energy and potentially inducing chemotherapy resistance (40). Our findings suggest that the impact of TP53 mutations may extend beyond the local tumor tissue. Given the central role of brown adipose tissue in energy metabolism and thermoregulation, TP53 mutations might systemically influence the metabolic function and status of adipose tissue, leaving distinctive metabolic signatures at the fat tissue level.

In this study, the model built on tumor PET radiomic features outperformed the CT-based model in accuracy, recall, and F1-score, demonstrating the superiority of PET imaging in reflecting tumor TP53 characteristics. Similarly, for brown adipose tissue radiomic features, the PET model showed more stable and overall better classification performance than the CT model, especially exhibiting outstanding accuracy and F1-score on the validation set. This indicates that metabolic information of brown adipose tissue is more important than its structural information for TP53-related features. The multimodal fusion model combining tumor and brown adipose tissue imaging features with pathological features further improved classification accuracy and robustness, showing consistently high performance on both validation and test sets. Notably, it achieved excellent recall, enabling more effective identification of positive samples and demonstrating strong generalization capability. After tumor annotation using an nn-UNet–based automatic segmentation model, the classification model’s performance was slightly lower than that with manual annotation but with a small gap. It also maintained a good balance between accuracy, precision, and recall, proving that automatic segmentation is feasible and stable for practical use, offering a fast alternative that does not require specialized medical imaging expertise.

The purpose of our study is not only to establish a model for tumor microenvironment recognition but also to extract features using a deep learning model and cluster them to reveal their correlation with cancer types. We hypothesize that a specific microenvironmental state, potentially characterized by multi-modal medical imaging biomarkers extracted through deep learning, can detect the same gene mutation across different tumor types. Recent studies have demonstrated the potential of radiogenomics in predicting the mutation status of genes like PD-1 and TP53 based on CT images and molecular markers (41). In previous artificial intelligence research, it is a mainstream practice to use single-modal data, which is conducive to building a more stable expert model (42, 43). However, these studies did not indicate whether specific gene mutations exhibit identical image features across different tumors. In this study, unsupervised clustering of features extracted by the deep learning classification model revealed that when the number of clusters was set to three, the Davies-Bouldin Index (DBI) reached its minimum value, significantly outperforming other cluster counts. This suggests the presence of three distinct imaging patterns closely associated with TP53 mutation status. Visualization via t-SNE dimensionality reduction showed clear clustering results, with clustering performance before and after dimensionality reduction being nearly identical, indicating that the deep features extracted by the model already possess strong discriminative ability in the high-dimensional space. This finding was further validated by correlation analysis, which demonstrated a significant association between the imaging cluster patterns and TP53 mutation status (*p* = 0.001), indicating that the model successfully captured TP53-related features common across different cancer types. Additionally, among the extracted deep features, many were directly related to TP53 mutations, suggesting these features have strong biological significance and potential clinical value. This research reveals greater potential for deep learning to accurately depict the tumor microenvironment and underscores the universality of the concept of genomics (44).

Our study has several limitations. First, this study is a single-center study; constructing a more robust classification model requires the inclusion of a larger population cohort and multiple centers. Second, PET/CT in our center was not used for screening but rather based on patients’ economic status and disease stage, the patients included in this study had more advanced tumor stages and poorer grades (45). Third, in tumor-related medical image research, pathological images are one of the most important research data types (46, 47). Predicting tumor gene mutations based on pathological images has been proven feasible (48). This study only utilized pathology-related features due to the complexity of standardizing pathological images, which may require the incorporation of additional generative networks (49, 50). Besides that, WSI technology can provide more comprehensive pathological information of tumors, which is helpful to improve the feature extraction capabilities of the model (51). For endometrial cancer, researchers predicted the molecular subtypes of endometrial cancer from hematoxylin and eosin-stained whole-slide images with an area under the curve of 0.874 (95% CI, 0.856–0.893) (52). While that study was successful, whole-slide images are difficult to prepare, costly, and challenging to apply clinically (53). To ensure the robustness of the features extracted in this study, we selected a fixed set of radiomic features as input, which to some extent limits the model’s ability to directly extract features from images. Meanwhile, although the features extracted by the deep learning model have been shown to be associated with TP53 characteristics, their relationship with intuitive radiological descriptors has yet to be established. Considering that the first-order features of brown adipose tissue contain relatively clear and interpretable information, this study did not extract higher-order texture or spatial features. The stability of such advanced features still needs to be validated in a larger cohort. Therefore, future attention should be focused on explaining neural networks to obtain more explainable diagnostic information (54). Meanwhile, the multimodal feature fusion method used in this study—direct concatenation of feature vectors—still has room for improvement (55). In addition, there is still a lack of public databases based on gynecological tumors, and we will continue to work on this (56).

## 5 Conclusion

This study confirms that radiomic features derived from CT and PET images of tumors and brown adipose tissue are closely associated with TP53 mutation status in gynecological tumors. By constructing multiple Transformer-based deep learning models and integrating them into a multi-modal combined model, we achieved high accuracy in predicting TP53 mutations and successfully captured cross-cancer imaging phenotypes significantly associated with TP53 status. The proposed cross-cancer TP53 prediction model offers a promising noninvasive tool for tumor molecular subtyping, with potential applications in personalized treatment planning, early risk stratification, and selection of targeted therapeutic strategies. This study highlights the potential of deep radiomics in bridging medical imaging and genomics, promoting the application of precision medicine in gynecological oncology.

## Data Availability

The raw data supporting the conclusions of this article will be made available by the authors without undue reservation.
